# Short-Chain Fatty Acids in the Metabolism of Heart Failure – Rethinking the Fat Stigma

**DOI:** 10.3389/fcvm.2022.915102

**Published:** 2022-07-11

**Authors:** Constantin L. Palm, Kirsten T. Nijholt, Barbara M. Bakker, B. Daan Westenbrink

**Affiliations:** ^1^Department of Cardiology, University Medical Centre Groningen, University of Groningen, Groningen, Netherlands; ^2^Department of Pediatrics, University Medical Centre Groningen, University of Groningen, Groningen, Netherlands

**Keywords:** heart failure, short-chain fatty acid (SCFA), fatty acid oxidation (FAO), metabolic reprogramming, cardiac fibrosis (CF)

## Abstract

Heart failure (HF) remains a disease with immense global health burden. During the development of HF, the myocardium and therefore cardiac metabolism undergoes specific changes, with decreased long-chain fatty acid oxidation and increased anaerobic glycolysis, diminishing the overall energy yield. Based on the dogma that the failing heart is oxygen-deprived and on the fact that carbohydrates are more oxygen-efficient than FA, metabolic HF drugs have so far aimed to stimulate glucose oxidation or inhibit FA oxidation. Unfortunately, these treatments have failed to provide meaningful clinical benefits. We believe it is time to rethink the concept that fat is harmful to the failing heart. In this review we discuss accumulating evidence that short-chain fatty acids (SCFAs) may be an effective fuel for the failing heart. In contrast to long-chain fatty acids, SCFAs are readily taken up and oxidized by the heart and could serve as a nutraceutical treatment strategy. In addition, we discuss how SCFAs activate pathways that increase long chain fatty acid oxidation, which could help increase the overall energy availability. Another potential beneficial effect we discuss lies within the anti-inflammatory effect of SCFAs, which has shown to inhibit cardiac fibrosis – a key pathological process in the development of HF.

## Introduction

Heart failure (HF) is one of the leading causes of hospitalization globally and is associated with substantial mortality. In addition to an overwhelming prevalence of more than 37.7 million people globally, the incidence is expected to grow further due to the increasing age of the world's population. Several common diseases like coronary artery disease and hypertension can cause this clinical syndrome, which is characterized by a growing inability of the heart to meet the body's demand in oxygen supply in form of circulating blood (1). The development of new HF drugs has made an important contribution to improving the life expectancy of patients with HF. However, these patients still suffer from a distinctly diminished quality of life, have reduced exercise capacity and 50% still die within 5 years of the initial diagnosis (2).

HF is defined as the inability of the heart to pump sufficient blood to meet the bodies energy requirements and can manifest itself with a number of symptoms such as dyspnea, fatigue and congestion. All cardiac diseases can ultimately cause heart failure, making cardiac dysfunction the final common endpoint of heart disease. Irrespective of the etiology the myocardium undergoes a number of pathological changes once HF has developed, characterized by cardiomyocyte hypertrophy, microvascular dysfunction, fibrosis and alterations in metabolism, often referred to as pathological cardiac remodeling (3). The heart is the organ with the highest energy turnover in the body which is required to sustain the circulation, continually converting chemical energy into mechanical work. It can only store enough energy to sustain three heart beats, making it exquisitely sensitive to disturbances in the supply of oxygen and metabolites. This is not only apparent from the clinical consequences of coronary artery disease. The development of all forms of HF is also associated with maladaptive changes in cardiac metabolism that eventually cause an energy deficit.

Most cardiac energy (60–90%) is generated by the oxidation of (long-chain) fatty acids (FA). The remainder is thought to primarily originate from carbohydrates. The heart is, however, a metabolic omnivore that alters its substrate use depending on prevailing supply and metabolic conditions. In HF, the mitochondrial capacity to oxidize FA and carbohydrates is diminished and ATP production is reduced (4). Although FA remain the main energy source for the failing heart, a “substrate switch" from FA toward glucose oxidation is thought to occur (5). Based on the dogma that the failing heart is oxygen-deprived and on the fact that carbohydrates are more oxygen-efficient than FAs, metabolic drugs for HF have so far aimed to either stimulate glucose oxidation or inhibit FA oxidation (4). Unfortunately, decades of clinical trials testing various compounds have not produced meaningful clinical benefits. We believe that it is time to rethink the concept that fats are bad for the heart. In this review, we will first introduce the metabolism of the healthy heart, subsequently that of the diseased, failing heart, and finally the mechanisms enabling fatty acids to potentially play a role in the treatment of HF in spite of their bad reputation.

## Cardiac Metabolism of the Healthy, Physiological Heart

As mentioned, the heart can switch between various substrates as sources of energy, and many adaptive metabolic changes occur during sustained exercise and pregnancy (6). The most dramatic change occurs during the transition from fetal to adult state in which glucose and lactate are the main cardiac energy sources, FA oxidation predominates thereafter (7). This switch toward FA oxidation is thought to be mediated by long-chain fatty acids (LCFAs) in breast milk, which activate the peroxisome proliferator-activated receptor-gamma coactivator-1α (PGC-1α) - peroxisome proliferator-activated receptor (PPAR) -α, -β, and -δ pathways as key transcriptional regulators of the genes responsible for FAO. This has several downstream effects, such as higher expression of fatty acid transporters, increased mitochondrial biogenesis and mitochondrial metabolism, leading to the described switch in substrate preference (8).

In the physiological adult heart of model organisms, ≈65% of ATP derives from FAO, the lion's share being long-chain fatty acids and ≈30% from oxidation of pyruvate, which is either the product of lactate oxidation or glycolysis, leaving only a minimal amount of ≈5% coming from anaerobic pathways (9–11). The metabolic benefit of this energetic switch is obvious, the net energy yield of long-chain FAO is 105 ATP molecules per molecule of palmitic acid, a common long-chain fatty acid. This is substantially higher than for a glucose molecule where oxidation results in 31 ATP per molecule or two ATP molecules for anaerobic glycolysis. The process of FAO is well-described, but a short overview of the steps of FAO is necessary to understand the changes in dysfunctional myocardium that are discussed below. Fatty acids enter the body via the digestive tract. While LCFAs and medium-chain fatty acids (MCFAs) require transporters to be taken up by enterocytes, short-chain fatty acids (SCFAs) can diffuse freely into the intestinal cells (12). Within the enterocyte, LCFAs and MCFAs together with lipoproteins form chylomicrons for transport. Chylomicrons are transported via the lymphatic system and enter the bloodstream via the thoracic duct. SCFAs are transported directly to the blood via the portal vein to the liver and are then released into systemic circulation (12). From the blood, SCFAs are taken up for further metabolization and oxidation by cardiomyocytes and other cell types of the heart.

The regulation of FA oxidation by cardiac myocytes is controlled at two regulatory hubs, the passage across the cell membrane and the passage across the mitochondrial membrane, although those differ depending on the chain length of the FA. LCFAs are taken up in a transporter-dependant manner. To enter the cell LCFAs bind to fatty acid translocase (FAT/CD36), which leads to internalization of both FAT/CD36 and LCFAs (13, 14). Inside the cell LCFAs are bound to carnitine to enable transport across the inner mitochondrial membrane by the carnitine shuttle consisting of carnitine palmitoyltransferase (CPT) 1 and 2. Since this is the rate-limiting step of FAO, CPT1 is considered the pacemaker enzyme of FAO (10, 15). MCFAs enter the cell via monocarboxylate transporter 1 (MCT1) and are also transported to the mitochondrial matrix by CPT1. MCFAs with a chain length of eight carbon atoms or less, can diffuse freely across the mitochondrial membrane (16, 17). SCFAs enter the cell via MCT1, or diffuse into the mitochondria in a transporter-independent manner. Their passage into the mitochondrion is also transporter-independent. This makes the utilization of SCFAs and MCFAs less susceptible to perturbations in transporter abundance and kinetics. However, oxidation of SCFAs is largely independent of the described transporters, which might provide the vital advantage over MCFAs in terms of oxidation rates, a fact which we will discuss in more detail later (18). Once inside the mitochondrion, all fatty acids are broken down through beta-oxidation, although the process is initiated depending on the length of the fatty acid by very-long-chain, long-chain, medium-chain and short-chain acyl-CoA dehydrogenase (VLCAD, LCAD, MCAD, SCAD) (19). The following steps of beta-oxidation are hydration, oxidation with release of electrons which enter the respiratory chain and release of an acetyl-CoA molecule which enters the Krebs cycle. This cycle is repeated several times, each time shortening the chain of FA by one molecule of acetyl-CoA; the longer the chain, the more cycles are required to degrade the FA (19). For an overview of the characteristics of LCFA, MCFA and SCFA (see Table 1).

**Table 1:** Overview of characteristics of different fatty acids according to chain length.

	**SCFA**	**MCFA**	**LCFA**
Carbon chain length	1–6	6–12	13–21
Common FAs in human context	Acetic acid (C2) Propionic acid (C3) Butyric acid (C4)	Caprylic acid (C8) Capric acid (C10) Lauric acid (C12)	Myristic acid (C14) Palmitic acid (C16) Stearic acid (C18)
Typical food containing FAs	- Dairy products - Vinegar - Fermentation of fiber*	- Dairy products - Palm oil - Coconut oil	- Dairy products - Palm oil - Olive oil - Meat

*SCFA, short-chain fatty acids; MCFA, medium-chain fatty acids; LCFA, long-chain fatty acids (20–23)*.

Despite its negative clinical stigma as a nutrient that leads to obesity and atherosclerosis when consumed in excess, fat is essential for proper cardiac function. However, effective FAO is only guaranteed under abundant supply of oxygen (9, 24). In response to repetitive endurance stress such as in swimming, the heart becomes physiologically hypertrophied. In addition to structural adaptations such as increased capillary density, enlargement of cardiomyocytes and a moderate increase in cardiac mass, cardiac metabolism also changes (25). Upregulation of PGC-1α -PPAR-α is responsible for an increase in mitochondrial fatty acid oxidation capacity – which leads to overall increased FA utilization and oxidation (26). Additionally, PGC-1α activation induces mitochondrial biogenesis, further contributing to the increase in oxidative phosphorylation (4, 26).

The heart is considered to be a metabolic omnivore, adapting its substrate use on the prevailing conditions and availability. In the physiological setting, however, most of its energy is derived from the oxidation of LCFA. In the next section, we will explain why in the context of HF passage across the cell membrane and transport into the mitochondria are so critical.

## Cardiac Metabolism in the Diseased, Pathological Myocardium

Overall oxidation of FAs has been shown to be decreased in HF. This has been demonstrated in rats with cardiac hypertrophy from pressure overload and in myocardial infarction, both of which resulted in a decrease in palmitate utilization (27, 28). Oxidation of oleate, another LCFA, is decreased in pacing-induced HF in dogs (29). The findings from animal data are consistent with human data, as patients with nondiabetic dilated cardiomyopathy (CM) and, in a second study, patients with idiopathic CM had decreased palmitate oxidation (30, 31). These changes are reflected by alterations in gene expression as well. MCAD expression was downregulated in the above-mentioned model of pacing-induced HF in dogs and an infarct rat model, so was long-chain fatty acid transporter FAT/CD36 in the infarction model (28, 29). CPT1 and 2, the crucial parts of the carnitine shuttle, are also downregulated in rodent HF models (27, 32). The reduction in fatty acid oxidation is at least partially mediated by the downregulation of PPAR-α and PGC1- α, which has been shown in a rodent HF models as well as in patients with both ischemic and non-ischemic end-stage HF (33, 34). In addition, the expression of transporters CPT1 and FAT/CD36 were found to be reduced in ischemic and dilated end-stage HF in model organisms and in humans as were the enzymes responsible for beta oxidation of very-long chain and long-chain FA, VLCAD, and LCAD. The latter mechanism is thought to be responsible for a “backlog” in FA metabolism, resulting in accumulation of toxic lipid intermediates in the heart, further aggravating the metabolic derangements in HF (32, 35, 36).

Even though the above-described studies are not consistent on all levels, there is overwhelming and consistent evidence that fatty acid oxidation is perturbed in HF. The reduction in FAO leads to an increase in anaerobic metabolism in the form of glycolysis in HF with an overall decrease in the available ATP (27, 37). This reduction in FA oxidation is considered fetal remodeling – although it must be mentioned that the metabolic differences are far less pronounced than between the adult and the fetal heart. The failing heart continues to yield 70% of its energy from FAO and it is questionable whether this should be considered a “metabolic switch” or rather a disruption in FA oxidation capacity. Of note glucose oxidation is also downregulated in advanced HF, indicating that a swich toward glucose oxidation is less likely. Nevertheless, the evidence does support the hypothesis, that the energy deficiency in the failing heart is caused by the inability to process long chain fatty acids (33, 38). The disappointing results from efforts to increase carbohydrate metabolism in heart failure, have led to the search for an alternative fuel for the failing heart. Recent work from our department and from other research groups have identified ketone bodies as a promising candidates, at least in part because they are taken up by cardiac mitochondria in a transporter-independent fashion (39, 40). Intriguingly, short-chain fatty acids (SCFAs), have similar bioenergetic properties as ketone bodies and appear to be even more efficient fuels.

## Short-Chain Fatty Acids (SCFAs) in a Healthy, Physiological Setting

**Figure 1:**
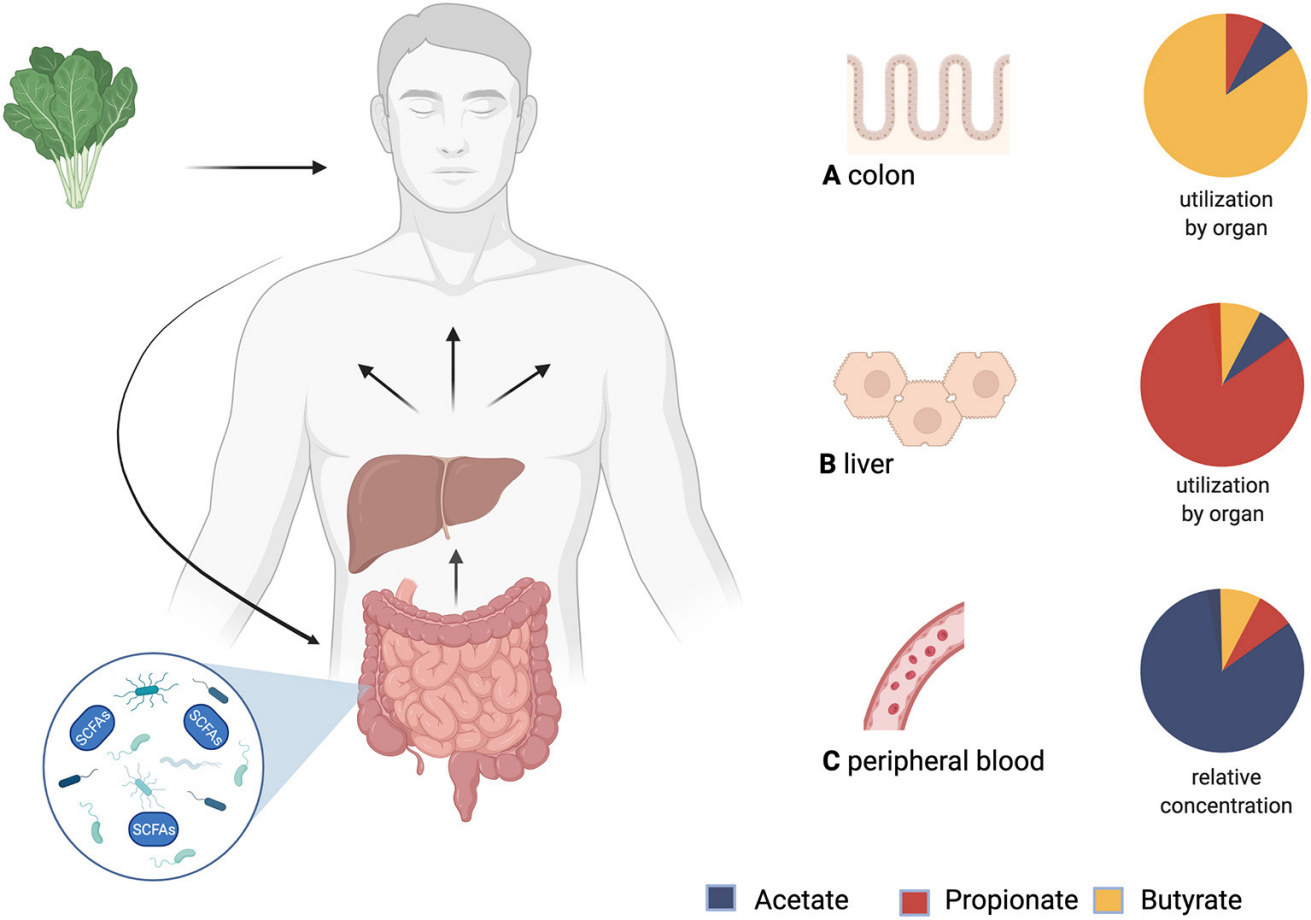
Production, distribution and yield of short-chain fatty acids (SCFAs) in different tissues. After consumption, undigested fiber reaches the colon and is fermented by microbiota. The product of this process are SCFAs. Via the portal vein SCFAs are transported to the liver and then systemically distributed. **(A)** Utilization of SCFAs in the colon: Butyrate is an important energy source for colonocytes. **(B)** Utilization of SCFAs in the liver: Propionate is mostly metabolized in the liver. **(C)** Relative concentrations of SCFAs in peripheral blood: Acetate has the highest peripheral concentrations. This is followed by propionate, while butyrate shows the lowest peripheral concentrations (43). Created with BioRender.com.

Fatty acids with a carbon chain of up to six atoms are considered SCFAs (41). The most relevant ones in human metabolism are acetate (C2), propionate (C3), and butyrate (C4). SCFAs originate from the fermentation of indigestible dietary fiber in the colon by the gut bacteria and are taken up by non-ionic diffusion and active transport into the colonocytes for which butyrate is the main source of energy. Propionate and acetate are transported via the portal vein into the liver, where propionate is further metabolized e.g., it serves as a substrate for gluconeogenesis, whilst acetate is found in higher levels in the periphery. Some butyrate also bypasses the liver by absorption in the distal colon (42) (see Figure 1). The contribution of SCFAs to total energy demand in healthy adults is around 10% (44).

It is well-known that a diet high in fiber is beneficial for health, people consuming high amounts of vegetables and fruits are less likely to develop obesity, diabetes mellitus and cardiovascular disease. However, the health promoting benefits spread much wider and also include protective effects for oncological diseases such as carcinomas of the colon, breast and liver (45). The majority of the beneficial effects of high-fiber diets could be linked to the production of SCFAs by the gut microbiome. In addition to serving as a fuel, SCFAs inhibit histone deacetylases (HDACs) which are known to promote inflammation and tumourigenesis (46, 47). The exact pathway of HDAC inhibition remains unclear. As of yet, direct inhibition of enzymatic activity and binding to G-protein-coupled receptors (GPCRs) are the two main proposed pathways. The main GPCRs to which SCFAs bind are GPR41, GPR43 and GPR109A (48, 49). SCFAs also protect against food allergies and colitis, and are even considered a potential drug for Alzheimer's disease and arthritis (50–53).

In rodent studies dietary supplementation with sodium butyrate prevented the usual development of insulin resistance and obesity in a high-fat diet – while food intake remained unchanged. This was thought to be the result of an increase in PGC-1α expression, which coincided with increased energy expenditure and oxygen consumption (54). A similar effect was observed with oral application of acetate, with improved insulin tolerance in mice with diabetes type II. It also lowered expression of lipogenic genes and abdominal fat mass. The authors attributed this effect to the increased phosphorylation of AMP-activated protein kinase (AMPK), which activates the PGC-1α signal pathway and thus inhibits fatty acid synthesis while increasing FAO (55). Butyrate as well-has been proven to increase phosphorylation of AMPK in adipocytes and has a stimulating effects on mRNA expression of PGC-1 and CPT1 in skeletal muscle (54, 56, 57). Acetate also influences apetite. Acute food intake in rats was lower following acetate injection by activation of hypothalamic neurons responsible for satiety (58). Oral intake of inulin-propionate ester promoted postprandial levels of satiating hormones peptide YY and glucagon-like peptide 1 and in the long term had anti-diabetic effects such as reduced weight gain, lower intra-abdominal adipose tissue mass and higher insulin sensitivity in a population of overweight humans (59). Distal colonic acetate infusion in obese men led to higher FAO rates, a fact the authors related partially to higher levels of satieting hormone peptide YY (PYY), but suggested might also be caused by an increase in AMPK like in the rodent setting (60).

## Short-Chain Fatty Acids in the Diseased, Pathological Myocardium

Regarding cardiovascular health, it is of note, that patients with cardiovascular diseases have an altered intestinal microbiome compared to healthy individuals. In the gut microbiome of patients with hypertension butyrate-producing bacteria are diminished and serum butyrate levels decreased (61). Also, the degree of hypertension negatively correlates with the amount of butyrate and acetate producing bacteria. This suggests that with increasing severity of hypertension the population of acetate and butyrate producers is further reduced. Furthermore, the microbiome of HF patients shows a decreased diversity, with diminished populations of butyrate-producing strains. Since the majority of SCFAs is derived from microbial fermentation, this implies that patients with HF and cardiovascular disease have a lower overall capacity to produce SCFAs (62). If that is the case, therapies aiming to restore the balance of the gut microbiome would improve the capacity to produce SCFAs. This could increase circulating serum levels of SCFAs, thereby increase the availability of nutrients for the failing heart.

**Figure 2:**
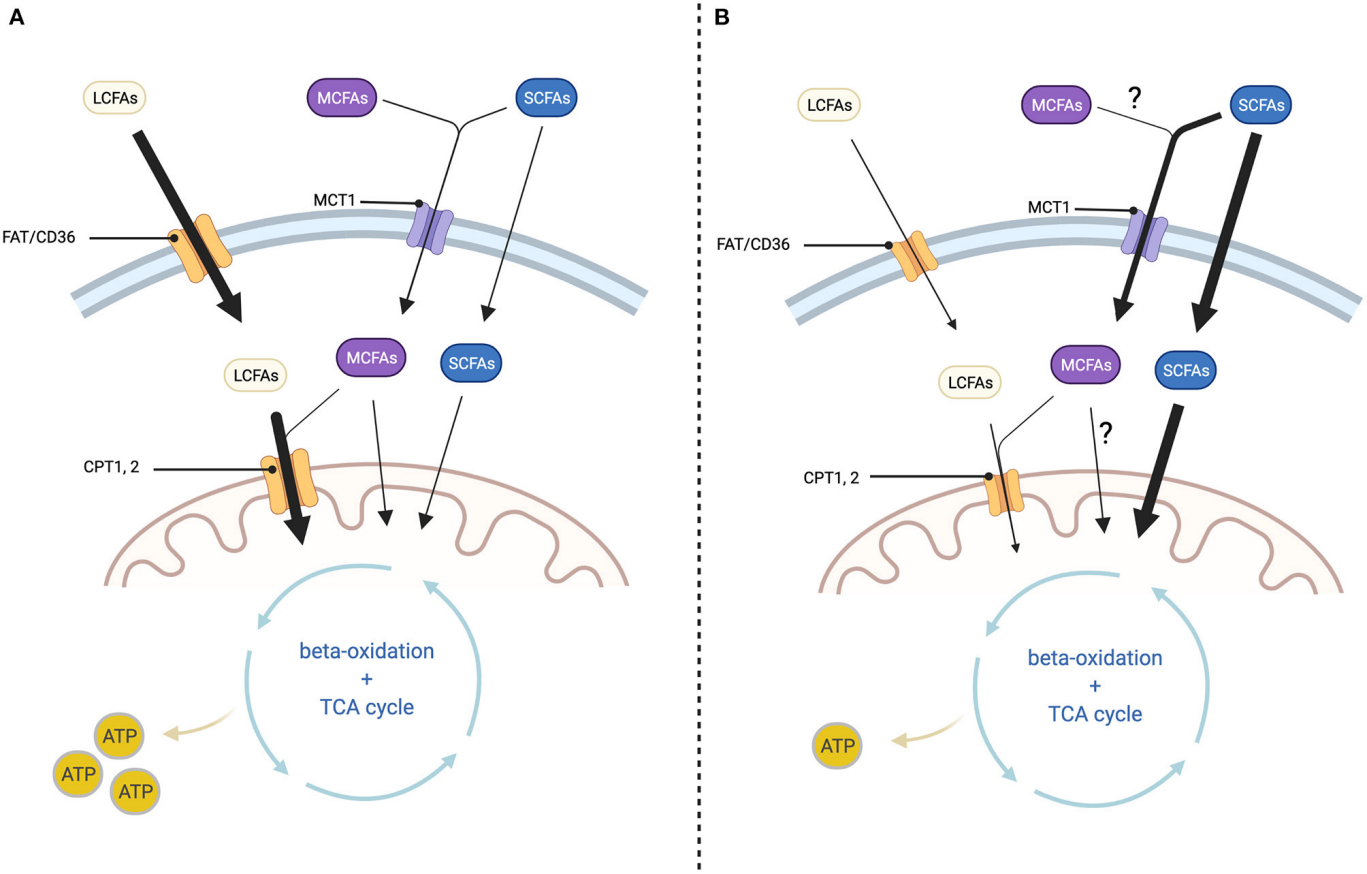
Cellular fatty acid transport depending on chain length and changes in HF. **(A)** In healthy myocardium: Long-chain fatty acids (LCFAs) enter the cell via fatty acid translocase (FAT/CD36) and the mitochondrion via the carnitine shuttle (CPT1, 2). Medium-chain fatty acids (MCFAs) enter the cell via monocarboxylate transporter 1 (MCT1) and the mitochondrion via CPT1, 2 or transporter independent. Short-chain fatty acids (SCFAs) enter the cell via monocarboxylate transporter 1 (MCT1) or through transporter-independent mechanisms. Passage across the mitochondrial membrane is as well-transporter-independent. Inside the mitochondrion both LCFAs, MCFAs and SCFAs are processed through beta-oxidation and the TCA cycle for ATP-generation. **(B)** In diseased myocardium: LCFA uptake into the cell and mitochondrion is decreased at least in part because the expression of FAT/CD36 and CPT1, 2 are diminished. On the other hand, SCFA uptake into the cell as well as the mitochondrion is increased, but this is not sufficient to prevent a reduction in ATP production. Changes in MCFA oxidation are not well-described. Created with BioRender.com.

Murashige et al. analyzed which nutrients the failing human heart uses to fulfill its energy demands. They found ketone, lactate and acetate metabolism to be increased. Even though the overall contribution of SCFAs to cardiac ATP production was relatively low, acetate extraction was increased by ≈20% in the HF cohort. Acetate uptake by the heart was also proportional to the circulating levels, suggesting that there is a large cardiac spare capacity for acetate oxidation that may be utilized for therapeutic purposes. In other words, increasing systemic SCFAs could be beneficial for energy production in HF (63). The increase in ketone as well as SCFA oxidation, is likely due to the fact, that their uptake into cardiac mitochondria is independent of CD36 and CPT1, enzymes that are downreglated in HF (18, 56, 64) (see Figure 2).

In an *ex-vivo* langendorff perfused heart model, Carley et al. (65) compared the oxidation rates of butyrate and the ketone body 3-OHB. Ketone body metabolism is increased in HF and substitution of ketones has been successfully tested as a strategy to improve myocardial function in HF mouse and rat models (39). Surprisingly, ATP yield from butyrate was significantly higher than from ketone bodies, both in healthy and failing hearts. They did not observe differences in metabolic regulation between substrates, suggesting that SCFAs are more efficient fuels than ketone bodies (65).

The same authors also evaluated further metabolic changes in human and rat HF. As expected, enzymes responsible for LCFA oxidation were reduced in failing hearts. Interestingly, however, they discovered a higher expression of acyl-CoA synthetase medium-chain family member 3 (ACSM3), an enzyme involved in butyrate oxidation (56, 66). ACSM3 is specific for fatty acids with carbon chains lenghts between 4 and 14 – this would make butyrate an ideal fuel candidate since it is a C4 fatty acid. Acetate and propionate would not be increasingly oxidized by this upregulation as they are no target substrates with C2 and C3 length. Considering that systemic acetate levels are higher than those of butyrate, further studies are necessary to clarify if one SCFA is more favorable as an energy source, or if even a combination of SCFAs might be beneficial.

As to our knowledge there are no reports of SCFAs activating AMPK in the myocardium. However, SCFAs have not been long investigated in the context of cardiac health, but effect similar to those described in other tissues (liver, skeletal muscle, brown adipose tissue) are conceivable (49, 54, 55). It would be interesting to evaluate whether AMPK activation by SCFAs takes place in the heart and if the extent differs between the individual SCFAs. This may have therapeutic potential, as AMPK is likely to be the major pathway of SCFA activation of PGC-1. As mentioned before, PGC-1 is a key regulatory pathway for FAO. By activating the PGC-1 pathway SCFAs might be able to at least partially restore the FAO capacities of the failing heart thus increasing its total ATP levels. Also there is indirect evidence suggesting SCFAs activate the PPAR-α -PGC-1 pathway in the myocardium. Crawford et al. (67) investigated the effect of fasting in germ-free mice and mice that had received a gut microbiome transplant. The physiological response to fasting would be an increase in PPAR-α expression leading to increased FAO and ketone body formation to maintain a constant energy supply. However, the germ-free mice had a markedly dimished expression of PPAR-α compared to those with a healthy gut microbiome. Considering the fact that SCFAs are known to increase PPAR-α in other tissues, it seems likely that the decreased PPAR-α expression in germ-free mice is caused by their lack of a SCFAs-producing microbiome.

The anti-inflammatory effect of SCFAs also plays a role in pathological cardiac remodeling. Tang et al. (68) altered the gut flora of mice by treatment with antibiotics to investigate the influence of gut microbiota on the postinfarction myocardial repair. They observed a significant increase in mortality in the antibiotic-treated mice, concomitant with a decrease in SCFA levels. The higher mortality was mitigated by 50% by giving SCFAs immediately prior to the infarction event. They attributed this effect mainly to an altered immune response taking into consideration the changes in a certain population of monocyte infiltration in the periinfarct zone. In terms of function, treatment with a probiotic cocktail containing Lactobacillus -a genus of bacteria known to produce SCFAs- was able to improve ejection fraction after the infarct event, although circulating SCFA levels remained unchanged. In a hypertension mouse model, Bartolomaeus et al. (69) demonstrated that oral treatment with propionate reduces development of atherosclerosis, but also reduces pathological cardiac hypertrophy and fibrosis. As the cardioprotective effects of propionate were less pronounced when given to a population of regulatory T cell-depleted mice, they suggested that propionate acts via a T cell-mediated pathway.

For a brief overview of studies that investigated SCFAs in the context of cardiovascular disease (see Table 2).

**Table 2:** Studies investigating the role of SCFAs in cardiovascular diseases.

**References**	**Title**	**Model**	**Findings**
Carley et al. (65)	Short chain fatty acids outpace ketone oxidation in the failing heart	- Rat - 14d after transverse-aortic constriction	SCFAs oxidation is increased in HF. SCFA are preferred over ketones as a fuel.
Lewandowski et al. (70)	Mitochondrial preference for short chain fatty acid oxidation during coronary artery constriction	- Pig - During coronary artery constriction	In hypoperfused myocardium, SCFA oxidation is increased.
Tang et al. (68)	Loss of Gut Microbiota Alters Immune System Composition and Cripples Postinfarction Cardiac Repair	- Mouse - Coronary artery ligation after 7d antibiotic treatment	SCFAs regulate myocardial repair after infarction.
Murashige et al. (63)	Comprehensive quantification of fuel use by the failing and nonfailing human heart.	- Human - HF with reduced EF and healthy hearts	Both HF and normal hearts rely in major parts on FA for energy. SCFA metabolism is increased in HF.
Bartolomaeus et al. (69)	Short-Chain Fatty Acid Propionate Protects from Hypertensive Cardiovascular Damage	- Apolipoprotein E knockout and WT mice - Angiotensin II infusion (14d and 28d) - oral propionate	Propionate treatment reduces cardiac hypertrophy and fibrosis, hypertension and atherosclerosis.

## Discussion And Future Perspectives

The fact that a high-fiber diet is health-promoting is well-described and this is at least partly due to the products of its fermentation, SCFAs. Long time they have only been recognized in the context of gut and bowel physiology, but their effect reaches farther than just the intestine. They have protective properties against the development of diabetes and oncological diseases and show antiinflammatory effects with possible application in the treatment of Alzheimer's disease and arthritis (52–54, 59).

But, most interesting to us, SCFAs also have a considerable potential as metabolic therapy in HF.

The abovementioned studies display SCFAs as a potent fuel since their oxidation is not dependent on the transporters of LCFAO, which are downregulated in HF (27, 65, 70). Thus, we suggest increasing systemic levels of SCFA might be an efficient way to improve cardiac function. A first step to evaluate the capability of SCFAs as energy source, would be an *in vivo* HF animal model on a diet (consisting of resistant starch/arabinoxylan oligosaccharides etc.) that leads to elevated systemic levels of SCFAs with cardiac function studies such as echocardiography, cardiac output and left ventricular pressure measurements. Analyzation of respiratory oxidation rates and determination of total ATP level in the heart would give insight on changes in mitochondrial function. A diet-based approach to increase the amount of circulating SCFAs has been established in humans in non-HF settings and would be to our knowledge safe to transfer to a human HF cohort (52, 71). However, for now, it is unclear if certain types of HF show different alterations in fatty acid metabolism and therefore deeper metabolomic insights are first necessary to identify the population most suitable for a human SCFAs trial.

In addition to the described fuel function, SCFAs might bear the potential to reverse the metabolic reprogramming in HF (72). Determination of expression levels of PGC-1 and related genes would help to clarify if the FAO enhancing effects of SCFAs are restricted to liver, skeletal muscle and fat tissue, or also take place in the heart.

The anti-inflammatory effect could be assessed by gene analysis of profibrotic pathways such as alpha-smooth muscle actin and connective tissue growth factor, but also by histological stainings (68, 73).

The derangements in fatty acid oxidation occurring in the failing heart may well-be of use in terms of diagnostics and prognostics. In the long term, SCFAs might be of use as biomarkers in HF, reflecting the metabolical state of the heart.

So far SCFAs show promising results and have the characteristics necessary to act as a metabolic therapy for the failing heart in several ways. Further research is needed in order to investigate their specific pathophysiological role and whether they could be a new therapeutic approach for HF.

## Author Contributions

CP, KN, BB, and BW were responsible for the article drafting and revising. All authors approved the final version of the manuscript, met the criteria for authorship, and all who qualify for authorship are listed.

## Funding

BW was supported by the Netherlands Organization for Scientific Research (NWO VENI, grant 016.176.147) and the Netherlands Heart Foundation Senior Clinical Scientist Grant (2019T064) and CVON DOUBLE DOSE (grant 2020-8005).

## Conflict of Interest

The UMCG, which employs KN and BW, has received research grants and/or fees from AstraZeneca, Abbott, Boehringer Ingelheim, Cardior Pharmaceuticals Gmbh, Ionis Pharmaceuticals, Inc., Novo Nordisk, and Roche. The remaining authors declare that the research was conducted in the absence of any commercial or financial relationships that could be construed as a potential conflict of interest.

## Publisher's Note

All claims expressed in this article are solely those of the authors and do not necessarily represent those of their affiliated organizations, or those of the publisher, the editors and the reviewers. Any product that may be evaluated in this article, or claim that may be made by its manufacturer, is not guaranteed or endorsed by the publisher.
